# Inpatient interventions that may preclude outpatient open pyeloplasty in infants

**DOI:** 10.1590/S1677-5538.IBJU.2018.0252

**Published:** 2019

**Authors:** M. İrfan Dönmez, Alonso Carrasco, Amanda F. Saltzman, Duncan T. Wilcox

**Affiliations:** 1 Department of Urology, Denver Anschutz Medical Campus, University of Colorado, CO, United States;; 2 Department of Pediatric Urology, Children's Hospital Colorado, Denver, CO, United States

**Keywords:** Ureteral Obstruction, Child, Anesthesia

## Abstract

**Objective:**

In the majority of published series, children undergoing open pyeloplasty are admitted for at least one night. We hypothesized that it would be possible in the majority of infants to perform open pyeloplasty as an outpatient procedure.

**Materials and Methods:**

All patients who underwent open pyeloplasty by a single surgeon between 2008 and 2016 were retrospectively reviewed. Demographic data (age at surgery, gender, pre- and postoperative imaging studies, laterality, type of local anesthesia), operative time, duration of hospital stay, need for narcotic analgesics, complications, readmission within 1-month after surgery and need for additional procedures were abstracted.

**Results:**

A total of 18 infants underwent open pyeloplasty by single surgeon. Mean age at time of surgery was 19 months (range 3-23 months). There were 8 girls and 10 boys. In addition to general anesthesia, all of the patients received regional anesthesia (caudal block 8, epidural block 8, subcutaneous nerve block 2). Median operative time was 135 minutes (range 81-166). Median hospital stay was 1 day (range 1 to 2). Two patients required iv narcotics for pain management. None of the patients required parenteral administration of other medications during the short hospitalization. No patients required any additional procedures or hospital readmissions within 1 month from surgery.

**Conclusions:**

In appropriately selected patients, outpatient pyeloplasty appears to be feasible with an oral postoperative analgesia plan to be administered at home.

## INTRODUCTION

Prenatal hydronephrosis complicates 1-5% of all pregnancies (1). Up to 88% of these cases may resolve spontaneously, with the remainder of patients requiring eventual surgical correction due to infection, loss of renal function, or worsening hydronephrosis (2, 3). Ureteropelvic junction obstruction (UPJO) is the most common cause of antenatal hydronephrosis, and thus is the most common condition requiring surgical correction (4). Surgical approaches for management of UPJO include open, laparoscopic and robot-assisted pyeloplasty, with comparable outcomes. Open pyeloplasty remains the gold standard due to its reproducibility.

Currently, infant pyeloplasty is generally done on an inpatient or overnight observation basis with most patients discharged the following day. However, there has been a recent move towards performing more pediatric surgeries on an outpatient basis (5, 6) because of the rapid recovery of children, low rates of complications associated with pediatric urologic procedures, and increased cost of inpatient care. Furthermore, this approach may help reduce anxiety of both the parents and infant after undergoing a surgical procedure (7).

Although there are some data regarding the feasibility of different outpatient surgeries in pediatric patients, no specific study has addressed pyeloplasty in infants (specifically those < 2 years of age at the time of surgery). The objective of the present study is to evaluate the feasibility of performing open infant pyeloplasty as an outpatient based on the professional medical care interventions required during postoperative hospital stay. We hypothesized that open infant pyeloplasty is associated with no major postoperative interventions which likely makes it feasible to be performed as an outpatient surgery.

## MATERIALS AND METHODS

After Institutional Review Board approval, all patients that underwent open pyeloplasty at < 2 years of age at the time of surgery by a single surgeon (DTW) between 2008 and 2016 were retrospectively reviewed. Demographic data (age at surgery, gender), pre- and post-operative imaging studies, laterality, type of local anesthesia, operative time, duration of hospital stay, need for narcotic analgesics, postoperative complications (infection, readmission, etc.), and need for additional procedures were reviewed. Postoperative care was specifically evaluated to determine the number of patients requiring professional medical care interventions that could not be accomplished at home and would thus necessitate an overnight stay postoperatively. Professional medical care interventions were defined as blood draws, urinary catheter insertion, procedures under anesthesia (ureteral stent placement, drain placement, reoperation), and use of intravenous drugs (opioids, antibiotics, anti-emetics) during the postoperative hospital stay. Initial recovery room interventions that could be available to patients as either inpatient or outpatient were excluded.

Surgical technique consisted of a 3 to 4 cm flank incision off the tip of the 11th or 12th rib using a muscle splitting technique. A dismembered pyeloplasty was subsequently performed in the standard fashion. No urethral catheter was inserted at time of procedure, and a Kidney Internal Split Stent (KISS) was used across the anastomosis to maintain drainage from the kidney in every case. The KISS stent was secured to the skin and left to gravity drainage for 24 hours and subsequently clamped. If this resulted in drainage around the stent or elicited discomfort it was placed back to gravity drainage and clamped again 24 hours later. The KISS stent was removed in clinic by postoperative day 7 if it had remained clamped for more than 48 hours without issue. No special dressings were applied and there were no food restrictions postoperatively. Patients received prophylactic antibiotics during the induction of anesthesia but not continued afterwards. For pain control, patients were placed on scheduled alternating acetaminophen and ibuprofen. All patients received local anesthesia consisting of either subcutaneous, epidural, or spinal nerve blocks at the discretion of the anesthesiologist.

Descriptive statistics are presented using non-parametric methods.

## RESULTS

A total of 18 infants who met criteria were identified. Indications for surgery were progression of hydronephrosis on serial renal ultrasonography or initially decreased or deterioration of ipsilateral kidney function on nuclear imaging studies. Median age at surgery was 19 months (range 3-23). There were 8 girls and 10 boys. A right pyeloplasty was performed in 7 patients, and a left pyeloplasty in 11. There were no cases of bilateral pyeloplasty. Median operative time was 135 minutes (range 81-166). Twelve patients were hospitalized for 1 day while 6 were discharged after 2 days. Median time of intra venous fluid administration was 17 hours (range 4-23 hours). Five patients received postoperative oral narcotics (oral oxycodone 0.05-0.1 mg / kg). However, just 2 patients (11%) required intravenous (iv) morphine (0.05 mg / kg), which necessitates a hospital stay (Table-1). Among the two patients who received IV analgesics, one had caudal block and the other had subcutaneous block. Also, both were < 12 months of age. For those patients requiring any narcotic pain control, median time from surgery to narcotic administration was 8 hours (range 6-22). No patient required parenteral administration of other medications during the postoperative hospitalization. No patients required any additional procedures and no patients required readmission within 30 days of surgery. No complications were observed. At follow-up of 3 months, 13 patients demonstrated improvement or resolution of hydronephrosis on renal ultrasound, 4 showed no change and 1 was lost to follow-up.

**Table 1:** Overview of the patients.

Number of patients		18
**Gender**		
	Male	10
	Female	8
Median age at surgery		19 months (range 3-23)
**Laterality**		
	Right	7
	Left	11
Median operative time		135 minutes (range 81-166)
Number needing oral narcotics (oxycodone)		5
Number needing professional medical intervention (iv narcotics)		2

## DISCUSSION

There is an increasing trend in pediatric urology towards performing more and more procedures on an outpatient basis. This is due to the rapid postoperative recovery and low complication rate associated with urologic procedures. Furthermore, in the era where cost effectiveness plays a major role in decision-making, all efforts are being focused on efficient and judicious use of resources without jeopardizing patient care and comfort.

Postoperative stay after pyeloplasty has been shown to be decreasing over time; 5 days in 1992 to 2 days in 2011 (8). This parallels the improved safety and feasibility of outpatient open urologic surgery (6, 9–11). Sprunger et al. reported 51 patients (mean age 4 years) who underwent outpatient procedures including ureteral reimplantation (n = 22), pyeloplasty (n = 20), hemi nephrectomy (n = 2), nephrectomy (n = 2), various ureteral reconstructions (n = 4) and pyelolithotomy (n = 1) via flank and / or Pfannenstiel incisions (6). Just 7 patients in the whole cohort were not discharged on the day of surgery and total postoperative hospitalization for these patients was 19.4 hours. Only 1 patient discharged the same day required urethral catheterization elsewhere. Even for flank incisions, which are typically very painful, 22 / 26 (85%) patients were discharged the same day as surgery. Mohamed et al. reported their experience with outpatient pyelolithotomy and pyeloplasty (514 patients with a mean age of 2.6 years). In their series, outpatient surgery was successfully performed in 437 (85%) patients (11). There were no observed complications requiring re-admission during the 3-year follow-up.

A possible limiting factor is the start time of the procedure. Patients should be in the OR as one of the first cases of the day to be able to be discharged during the day, which may have a negative impact on operation room planning. Current practice at this institution has the youngest patients as the first cases of the day, so these patients who are all < 2 years of age are likely occurring earlier as such. Also, families who live long distances from the hospital may not be suitable for performing outpatient pyeloplasty due to the burden of a long drive and remote access to care should it be needed postoperatively. Additionally, patients who clinically require close follow-up after surgery (difficulty in oral intake after surgery, irritability etc.) likely require postoperative admission for monitoring and the use of outpatient status may need to be abandoned. While median intravenous fluid administration was 17 hours in our study, this is highly variable and dependent on nursing preferences, thus it is not an objective surrogate for assessing oral intake tolerance.

After open pyeloplasty, just 10% of infants required inpatient postoperative care, exclusively in the form of pain management. Factors contributing to successful pain management include use of local blocks along with external drains (12). The current protocol at this institution includes preemptive analgesia in all patients in the form of caudal, epidural or subcutaneous nerve block, percutaneous nephroureteral stent, and early oral medications (acetaminophen, non-steroidal anti-inflammatory drugs, and oral narcotics when necessary). Although limited by small patient series, the present study did not identify that any patient required major interventions (i.e. catheter placement, exploration etc.) or early re-admission.

The psychological burden of surgery on patients and parents must also be considered. Hospitalization increases anxiety not only in pediatric patients but also in families (13). Even short duration hospitalization (2-4 days) may result in behavioral sequelae in children (14). Moreover, minor emergency procedures may have a negative impact on children's behavior in the short term manifesting as separation anxiety, aggression against authority, and sleep disorders (15). By decreasing the time infants spend in the hospital, the stress and anxiety experience may be reduced which can then minimize associated psychological side effects. However, we consider outpatient surgery in infants relies upon adequate preoperative parental counseling and cooperation, a well-functioning hospital system, adequate postoperative analgesia plan, and minimizing external drainage tubes, which can be easily managed by parents.

Limitations of this study include retrospective design, single center, single surgeon and a limited number of cases. Although these procedures were not performed as outpatient, based on our review of documented professional medical care interventions, there were only 2 interventions in the form of IV narcotic administration that could have not been performed by parents at home. Also, it should be noted that nurses had wide latitude to administer pain medications. With the current use of electronic medical records and strict documentation required by nursing staff and physicians for major interventions, it is unlikely that major interventions were missed during our retrospective review, however it is possible. This single surgeon, single center data regarding open dismembered pyeloplasty in infants supports the feasibility of performing this procedure as outpatient.

## CONCLUSIONS

After open pyeloplasty, infants require minimal professional medical care interventions, and this consists primarily of IV narcotic analgesia. Given the minimal interventions required during the postoperative hospitalization, performing this procedure on an outpatient basis appears to be feasible. A prospective trial is necessary to confirm the feasibility of outpatient pyeloplasty in infants.

### Compliance with Ethical Standards

This study was conducted according to the Declaration of Helsinki and upon the approval of local Institutional Review Board.

Informed consent: Informed consent was not obtained, as this is a retrospective study.
